# High Dietary Fructose: Direct or Indirect Dangerous Factors Disturbing Tissue and Organ Functions

**DOI:** 10.3390/nu9040335

**Published:** 2017-03-29

**Authors:** Dong-Mei Zhang, Rui-Qing Jiao, Ling-Dong Kong

**Affiliations:** State Key Laboratory of Pharmaceutical Biotechnology, School of Life Science, Nanjing University, Nanjing 210023, China; zdm@nju.edu.cn (D.-M.Z.); jiaorq@nju.edu.cn (R.-Q.J.)

**Keywords:** high dietary fructose, metabolites, metabolic syndrome, insulin resistance, oxidative stress, inflammation, tissue and organ dysfunction

## Abstract

High dietary fructose is a major contributor to insulin resistance and metabolic syndrome, disturbing tissue and organ functions. Fructose is mainly absorbed into systemic circulation by glucose transporter 2 (GLUT2) and GLUT5, and metabolized in liver to produce glucose, lactate, triglyceride (TG), free fatty acid (FFA), uric acid (UA) and methylglyoxal (MG). Its extrahepatic absorption and metabolism also take place. High levels of these metabolites are the direct dangerous factors. During fructose metabolism, ATP depletion occurs and induces oxidative stress and inflammatory response, disturbing functions of local tissues and organs to overproduce inflammatory cytokine, adiponectin, leptin and endotoxin, which act as indirect dangerous factors. Fructose and its metabolites directly and/or indirectly cause oxidative stress, chronic inflammation, endothelial dysfunction, autophagy and increased intestinal permeability, and then further aggravate the metabolic syndrome with tissue and organ dysfunctions. Therefore, this review addresses fructose-induced metabolic syndrome, and the disturbance effects of direct and/or indirect dangerous factors on the functions of liver, adipose, pancreas islet, skeletal muscle, kidney, heart, brain and small intestine. It is important to find the potential correlations between direct and/or indirect risk factors and healthy problems under excess dietary fructose consumption.

## 1. Introduction

The World Health Organization (WHO) defines metabolic syndrome (MetS) as a cluster of symptoms with impaired glucose tolerance or insulin resistance, together with two or more of the following components: raised arterial pressure, raised plasma triglyceride (TG) and/or low high-density lipoprotein (HDL) cholesterol, central obesity and microalbuminuria [1]. Central obesity and insulin resistance are acknowledged as the important causative factors in the pathogenesis of MetS [2]. MetS increases the risk of developing hypertension, cardiovascular disease (CVD), type 2 diabetes (T2DM), non-alcoholic fatty liver (NAFLD), hyperuricemia, gout and chronic kidney disease (CKD) [3].

Fructose is a monosaccharide found in fruits, vegetables and honeys. As for its sweetness, palatability and taste enhancement, fructose is widely added to processed food and beverages. High-fructose corn syrup (HFCS) is one of the most widely used food ingredients in nearly all soft drinks, canned jams, breakfast cereals and baked goods. High fructose diet and extensive commercial use of HFSC are reported to be associated with the rising prevalence of MetS worldwide [4], triggering function impairment in multiple tissues and organs. The metabolism of fructose is quite different from glucose in catabolic reaction, as well as metabolite and regulatory mechanism. Fructose is metabolized in liver via fructolysis, and the primary metabolites and by-products include glucose, lactate, free fatty acid (FFA), very low-density lipoprotein (VLDL)-TG, uric acid (UA) and methylglyoxal (MG). Extrahepatic absorption and metabolism of fructose also take place. These metabolites are considered to be direct dangerous factors, with the potential to disturb functions of extrahepatic tissues and organs.

In addition to rapid fructolysis in liver, high fructose causes an ATP depletion that triggers inflammatory response and oxidative stress, thereby disturbing functions of local tissues and organs. Subsequently, inflammatory cytokine, adiponectin, leptin, and endotoxin are produced and become indirect dangerous factors. Fructose and its metabolites directly and/or indirectly cause insulin resistance, chronic inflammation, endotoxin secretion, autophagy and disturbance of appetite for food intake, aggravating MetS.

Therefore, we will delineate fructose-induced tissue and organ dysfunctions resulting from these direct and/or indirect dangerous factors. It will focus on the correlations between different metabolites and functional assignment of different tissues and organs in the whole body under high fructose condition.

## 2. Absorption and Metabolism of Fructose

Fructose is directly absorbed across the brush border of the small intestine into enterocyte by glucose transporter 5 (GLUT5) [5], and transported out of the enterocytes into systemic circulation by GLUT2, located at the basolateral pole [5]. The transported fructose is delivered into the systemic circulatory system and absorbed mainly in liver.

More than 50% of fructose is metabolized via fructolysis in liver. Fructokinase (KHK) catalyzes the first phosphorylation reaction to produce fructose-1-phosphate (fructose-1-P) and initiates fructose catabolism [6]. Aldolase B catalyzes the lysis of fructose-1-P to generate dihydroxyacetone phosphate (DHAP) and glyceraldehyde, two major components of triose-Ps. DHAP and glyceraldehyde are converted to glucose following the conventional gluconeogenesis. Part of DHAP and glyceraldehyde are converted into lactate and released into circulation, others can be reversibly metabolized to glycerol-3-phosphate (glycerol-3-P), and catalyzed by glyceraldehyde kinase. MG synthase catalyzes glycerol-3-P and/or DHAP to produce MG, which is secreted into circulation. Meanwhile, glycerol-3-P forms FFA and TG via de novo lipogenesis (DNL). Diacylglycerol (DAG), an active lipid intermediate, is produced during TG generation. Then TG is packed with apolipoprotein B100 (ApoB100), facilitating VLDL-TG production and secretion. Glyceraldehyde can also be converted to acetyl-CoA (consecutively catalyzed by glyceraldehyde-3-phosphate dehydrogenase, phosphoglycerate kinase, phosphoglycerate mutase, enolase, pyruvate kinase and pyruvate dehydrogenase), either producing FFA, or further participating in TG synthesis or entering tricarboxylic acid cycle (TCA) cycle. Another bioactive lipid intermediate, ceramide, is derived from metabolism of palmitic acid (the preliminary products of FFA synthesis). Rapid fructolysis leads to a high level of metabolic stress via ATP depletion [7], increasing AMP degradation to increase UA in liver, finally resulting in blood UA elevation. Therefore, glucose, lactate, FFA, TG, VLDL-TG, DAG, ceramide, UA and MG are overproduced and released into systemic circulation. Some of them, such as glucose, lactate, FFA, VLDL-TG, UA and MG, are delivered to extrahepatic tissues, affecting energy hemostasis or impairing tissue and organ functions. High levels of these metabolites are considered to be direct dangerous factors under high fructose condition.

Extrahepatic absorption and metabolism of fructose also take place, since GLUT5 is also widely expressed with high specificity in adipose tissue, kidney, muscle skeletal tissue, testis and brain [5]. GLUT2, a low-affinity fructose transporter, is also located significantly in kidney and small intestine [5]. It is likely that maximal physiological, postprandial concentration of fructose reaches to 1.0 mmol/L in the portal vein, and remains in the micromolar range in peripheral blood in humans and rodents [6]. Plasma fructose concentration reaches up to 1–2 mM after the intravenous fructose infusion (22 mumol·kg^−1^·min^−1^) [8], but the half-life is about 20 min in normal subjects [9]. Therefore, extrahepatic fructose uptake does not occur to a significant extent due to its very low blood level.

During the fructolysis, a high level of metabolic stress via ATP depletion is detected [7]. ATP depletion causes oxidative stress and inflammatory response to disturb the function of tissues and organs, resulting in abnormal production of insulin, inflammatory cytokine, adiponectin, leptin and endotoxin. These indirect dangerous factors are secreted into systemic circulation, further aggravating metabolic burden in tissues and organs and even perturbing appetite and food intake.

## 3. Direct Dangerous Factors under High Fructose Consumption

### 3.1. Glucose

The impact of high fructose consumption on fasting glucose level is controversial and dependent on the differences in age, energy status, and drug dose during the experiments. High fructose intake produces immediate change in hepatic and extrahepatic substrate metabolism, but the overall glucose production remains unchanged in some reports of human subjects [10] and rodents [11]. More frequently, fasting or postprandial glucose concentrations are increased after high fructose consumption in clinical trials [12] and animal experiments [13]. The elevated glucose output may cause an increase of insulin demand and trigger insulin over-release.

Increased hepatic gluconeogenesis and glucose export are considered as the major causes of systemic insulin resistance [10]. Meanwhile, fructose-induced pancreatic β-cell dysfunction causes insulin secretion as well, via activating sweet taste receptor (TR) signaling in humans and mice [14]. Impairment of β-cell mass and function inmales with high fructose diets result from dysregulation of leptin signaling and activation of protein kinase B (PKB/Akt)/Forkhead box protein (Fox) O1 in rat islets [15]. ER stress occurs in pancreatic β-cells under high fructose diet, as it is closely associated with insulin resistance, inflammation and abnormal lipid metabolism, possibly leading to glucose intolerance and insulin resistance [13].

### 3.2. Lactate

High fructose increases postprandial lactate level, leading to hyperlactatemia [16]. Excessive pyruvate produced by fructolysis undergoes glycolysis almost completely in liver, exporting lactate into peripheral tissues and organs. Adipose [17] and skeletal muscle [18] can also produce lactate. In adipocytes differentiated from human Simpson-Golabi-Behmel Syndrome (SGBS) preadipocytes, fructose triggers the conversion of glucose to lactate, causing lactate release [19].

Approximately 40% of the released lactate is absorbed in skeletal muscle, and then oxidized or converted to glucose. Thus, lactate elevation in blood leads to systemic insulin resistance, and can be considered an independent risk factor for the development of T2DM [20]. Lactate infusion induces insulin signaling impairment by inhibiting phosphatidylinositol 3-kinase (PI3K) and Akt activity in skeletal muscle of mice [21]. On the other hand, lactate suppresses hexokinase (HK) and phosphofructokinase (PFK) in skeletal muscle, liver, heart and kidney, resulting in glucose consumption reduction [22]. Meanwhile, glucose uptake is reduced by hyperlactatemia via suppressing GLUT4 to decrease glycolytic flux in skeletal muscle [23,24]. Glycolytic flux inhibition by high lactate even happens in the presence of insulin [25]. Mice with over-expression of GLUT4 in skeletal muscle improve insulin sensibility and glucose uptake [24]. Therefore, fructose-induced high serum lactate may mainly target skeletal muscle to reduce glucose consumption and flux, resulting in systemic insulin resistance.

Of note, a high lactate level can reduce oxygen availability and enhance inflammatory response. Hypoxia is the crucial event for glycolysis acceleration and lactate release into systemic circulation. In both humans and murine adipocytes, hypoxia inhibits insulin signaling in a hypoxia-inducible factor (HIF)-1-dependent manner by decreasing insulin receptor (IR) phosphorylation and suppressing PKB, Akt, substrate-160 kDa (AS160) and GLUT1 in response to insulin [26]. Excessive oxygen consumption correlates with the impairment of insulin-stimulated glucose uptake, which may result from the upregulation of tribbles homolog 3 (TRIB3), a negative modulator of Akt, in skeletal muscle of rats with excess nutrients [27]. Meanwhile, interleukin (IL)-1β and IL-6 production and secretion are induced by hypoxia in adipocytes [26], which are known to trigger systemic and local insulin resistance [28]. Therefore, fructose-driven lactate overproduction is another event causing systemic and/or local insulin resistance.

### 3.3. Free Fatty Acids (FFAs)

High fructose consumption gives rise to hyperlipidemia [29]. Significant increase in hepatic DNL is one of the major adverse causes for metabolic burden under high fructose consumption. Therefore, increased plasma FFAs, TG and VLDL-TG levels induce hyperlipidemia, as well as TG accumulation in extrahepatic tissues and organs. Fructose-induced lipotoxicity leads to NAFLD, lipid accumulation and autophagy in skeletal muscle [30], cardiac dysfunction [31], adipose inflammation [32], CKD [33], pancreatic islet dysfunction [34], brain oxidative stress and inflammation [35].

Among the products and lipid metabolites by DNL, FFAs are the initial and primary risk factors for insulin resistance in liver and extrahepatic tissues and organs under high fructose diet. Adipose tissue, acting as a highly active metabolic and endocrine-producing organ, can also increase FFA secretion. It is reported that fructose (0.1–10 mM) directly stimulates de novo FFA synthesis in human SGBS pre-adipocytes [19]. Hepatic insulin signaling modulates glucose output to maintain serum glucose hemostasis through activating insulin receptor substrate (IRS)-1/2/PI3K/Akt pathway [11]; this pathway impairment causes hyperglycemia and compensatory hyperinsulinemia, cooperatively preceding systemic insulin resistance. Fructose feeding may upregulate hepatic carbohydrate response element binding protein (ChREBP) to activate glucose-6-phosphatase (G6Pase) and enhancing glycolytic flux, thus impairing glucose homeostasis [36].

Change of plasma FFA pattern closely links with systemic insulin resistance under high fructose diet. Reduction of plasma polyunsaturated FFAs, such as docosapentaenoic acid and docosahexaenoic acid, is closely associated with systemic insulin resistance induced by high fructose consumption [37]. Upregulation of ChREBP also activates stearoyl-coenzyme A desaturase (SCD)-1 to increase monounsaturated fatty acids (palmitoleic acid and oleic acid) and polyunsaturated fatty acids (PUFA, *n*-6 and *n*-3 polyunsaturated fatty acids) production in liver, which may account for hepatic insulin resistance in fructose-fed rats [11]. FFAs stimulate the intracellular translocation of Bcl-2-associated X protein (Bax) to the lysosome in hepatocytes, and consequently release cathepsin B, which inhibits insulin signaling by activating nuclear factor kappa B (NF)-κB to enhance tumor necrosis factor (TNF)-α secretion, leading to NAFLD [38]. Activation of a series of kinases, including protein kinase C (PKC)-θ, IκB kinase-β (IKK-β), c-jun *N*-terminal kinase (JNK) and S6-kinase may play a crucial role in insulin resistance induced by FFAs and the derived metabolites (DAG, ceramides and TG) [39]. These kinases can disturb serine phosphorylation of IRS and inhibit insulin signaling in hepatic or extrahepatic tissues and organs, such as white adipose tissue (WAT) [40] and skeletal muscle [41]. Some phosphatases also participate in fructose-induced insulin resistance. It is reported by us that fructose impairs hepatic insulin signaling by activating protein tyrosine phosphatase-1B (PTP-1B) in rats [42].

Pancreatic islet β-cell dysfunction under high fructose consumption is another adverse effect of lipotoxicity, giving rise to systemic insulin resistance. FFAs (palmitate or oleate:palmitate = 2:1) critically impair insulin secretion in isolated islets from humans [43] or C57BL/6 mice [44]. Intralipid feeding inhibits glucose-induced insulin secretion in rats, while long-term exposure of FFAs (palmitate, oleate or octanoate) further increases the ratio of proinsulin:insulin in isolated human islets exposed with fructose [45]. The dysfunction of pancreatic islet possibly results from downregulation of sterol regulatory element-binding protein (SREBP)-1c-mediated IRS-2/Akt pathway [44]. Palmitate also impairs glucose-stimulated insulin secretion and β-cell function in rat insulin-secreting INS-1 cells [46] and induces H_2_O_2_ formation in the peroxisomes of RINm5F insulin-producing cells [47], showing its lipotoxicity in vitro. Furthermore, palmitate exposure may induce apoptosis in isolated islets from humans [43,48], possibly through its receptor cell death-inducing DFF45-like effector b (Cideb) [48]. Oxidative stress is another negative effect under palmitate exposure, accompanied with apoptosis in βTC6 cells (a glucose-sensitive mouse β pancreatic cell line) through activating free fatty acid receptor 1 (FFAR1) [49]. Therefore, fructose-induced FFA production (especially palmitate) may disturb insulin secretion by disturbing the function of pancreatic islet β cells. High fructose consumption increases hepatic DAG level [50,51] and membrane-associated PKC activity [52,53], possibly resulting in insulin resistance. High DAG and ceramide concentrations are considered as disposal of excess FFAs in liver and cause insulin resistance. Parallel lipidomics analysis of liver tissues from mice and humans shows that DAG increase is suggested to be a hallmark of NAFLD [54]. DAG activates PKC through specific binding to PKC and promotes PKC translocation [55], suppresses Akt2 to decrease glycogen synthesis by inhibiting glycogen synthase (GS) and increases gluconeogenesis by activating G6Pase and phosphoenolpyruvate carboxykinase (PEPCK), leading to glucose release through GLUT2 in liver [56]. Fructose significantly suppresses carbohydrate utilization in mitochondria [50,51], contributing to insulin resistance in liver. PKC activation is also detected in adipose tissue, which may be associated with fructose-induced hypertriglyceridemia [52].

Fructose supplementation increases de novo ceramide biosynthesis and elevates ceramide concentrations in plasma [57], liver [58] and skeletal muscle [59], promoting local insulin resistance. Ceramide decreases the ability of insulin to activate Akt and GLUT4 translocation in 3T3-L1 adipocytes [60]. Ceramidases catalyzes ceramide to produce sphingosine, which may participate in insulin signaling impairment. The phosphorylation of sphingosine by sphingosine kinase (SphK) 1 produces sphingosine-1-phosphate (S1P). Our group shows that high fructose consumption induces SphK1/S1P signaling to activate NF-κB pathway, which accounts for lipid accumulation, insulin and leptin resistance, as well as inflammation in rat liver tissue [61]. Thus, SphK1/S1P signaling impairment is relevant to the development of MetS.

FFA overproduction mediates mitochondrial dysfunction, which may be another risk factor for insulin resistance under high fructose consumption. In different tissues of fructose-fed animals, mitochondrial dysfunction is detected, characterized by increased mitochondrial mass [62,63], decreased mitochondrial electron transport capacity [62,63], loss of mitochondrial membrane potential [64] and disturbance of antioxidant defense [32]. In turn, insulin resistance affects FFA-mediated mitochondrial uncoupling [65]. Meanwhile, decreased oxidative capacity resulting from mitochondrial impairment further suppresses FFA oxidation. Overproduction of reactive oxygen species (ROS) therefore causes insulin resistance under high fructose consumption [62,63]. Fructose consumption decreases hepatocyte NADPH oxidase 4 (NOX4) to elevate ROS production by reducing protein phosphatase 1c (PP1c) to impair insulin signaling [66]. Recently, we find that oxidative stress induces cardiac inflammation and fibrosis via scavenger receptor (CD36)-mediated toll-like receptor 4 (TLR4)/6-IL-1R-associated kinase 4/1 (IRAK4/1) signaling to suppress NOD-like receptor superfamily, pyrin domain containing 3 (NLRP3) inflammasome activation in fructose-fed rats [67]. Also, superoxide generation induced by high fructose diet increases blood pressure and blocks central insulin signaling [68].

Increased FFA uptake and ectopic deposition in extrahepatic tissues and organs, such as skeletal muscle, liver, pancreas islet and cardiovascular tissue, may result in lipotoxicity and insulin resistance under high fructose consumption. FFAs can be uptaken by several tissues via FFA transport proteins (FATPs) and CD36, both highly expressed in heart, adipose tissue, and skeletal muscle [69]. Fructose upregulates CD36 expression in adipose tissue [70] and skeletal muscle [71] to facilitate FFA uptake, resulting in local insulin resistance. Increased FFAs promote autophagy in skeletal muscle of mice with high fructose diet, likely as a compensate mechanism for clearance of lipotoxic intermediates [30].

Recently, fructose has been reported to be metabolized in several regions of brain, including cerebellum, hippocampus, cortex, and olfactory bulb, which express GLUTs and all of the enzymes in fructolysis [72], probably leading to central inflammation response. FFA elevation in plasma gives rise to hippocampal insulin signaling impairment and inflammation under high fructose consumption, since FFAs may cross the blood–brain barrier [73]. Hypothalamus is the major site sensing energy status in the whole body. The possible mechanism relates to neuropeptides secretion via regulation of AMP-activated protein kinase (AMPK) signaling and malonyl-CoA concentration, compensating for the change in energy status [74]. Rapid fructolysis results in ATP depletion to produce more AMP. Sensing increase of the AMP/ATP ratio, AMPK is activated under high fructose consumption [75]. Moreover, peripheral indirect signals generated by fructose, including TNF-α [76] can also activate AMPK in hypothalamus. Fructose triggers AMPK/malonyl-CoA signaling in hypothalamus, subsequently increasing food intake and the risk of obesity [77]. Furthermore, fructose-induced hypothalamic AMPK activation increases hepatic gluconeogenesis by the elevation of circulating corticosterone level, further contributing to systemic insulin resistance [78].

High fructose consumption gives rise to the development of cardiovascular disease by increasing VLDL-TG, TG, cholesterol, VLDL-cholesterol and low-density lipoprotein (LDL)-c, as well as decreasing HDL in circulation [79,80,81,82]. Overproduction and secretion of VLDL-TG under high fructose consumption is proposed to be the early markers of cardiovascular metabolic diseases [79], in which hepatic DNL induction through activation of SREBP-1c plays an important role [80]. Meanwhile, elevated plasma proprotein convertase subtilisin/kexin type (PCSK) 9 induced by fructose directly influences plasma LDL-C by downregulating hepatic LDL receptor (LDLR) expression [81]. The dysregulation of PCSK9/LDLR signaling induced by fructose may cause hypercholesterolemia, possibly playing a vital role in the development of atherosclerosis [82]. Palmitic acid, the main product of FFA synthesis, is reported to increase plasma cholesterol and LDL concentrations by suppressing LDLR in liver [83], thereby increasing the risk of atherosclerosis. Increased apoC-III, one of the components of VLDL in circulation, is observed under high fructose diet [84]. Elevated apoC-III induces hypertriglyceridemia [85] and insulin resistance [86], acting as another emerging pro-atherosclerosis factor. Moreover, apoC-III production senses FFA elevation in plasma [86]. Hepatic scavenger receptor class B type I (SR-BI) acts as HDL receptor, mediating HDL transport to liver. It decreases circulating cholesterol level and has atheroprotective action [87]. Fructose feeding increases intestinal SR-BI level and basal ERK activation (downstream of MAP kinase), accounting for local tissue insulin resistance, apoB48-invloved chylomicron assembly and overproduction [88]. Therefore, under high fructose consumption, elevated FFAs and the derivate DAG and ceramide contents in circulation result in insulin resistance in liver or other tissues and organs, while increased assembly form of DNL products is responsible for cardiovascular diseases.

### 3.4. Uric Acid (UA)

Uncontrolled fructose catabolism in liver induces rapidly ATP depletion to overproduce UA in systemic circulation, developing hyperuricemia in humans and experimental animals [89]. Epidemiological studies reveal that hyperuricemia has close relationship with insulin resistance, inducing gout, hypertension, atherosclerosis and chronic renal diseases [90]. Elevated UA may exacerbate DNL by stimulating hepatic KHK [91] and lipogenic enzymes [92], further aggravating fatty liver. Fructose-induced serum UA elevation is responsible for ROS generation in liver [92] and extrahepatic tissues, including adipose tissue [93], skeletal muscle [94] and aorta [95]. Consequently, local oxidative stress promotes hepatic steatosis [92], skeletal oxidative stress [94], cardiac hypertrophy [96], and kidney dysfunction [97].

UA-promoted oxidative stress under high fructose consumption triggers inflammatory response, including secretion of TNF-α, IL-1β, transforming growth factor (TGF)-β1 and monocyte chemotactic protein (MCP)-1 in kidney [98,99]. Thus, renal NLRP3 inflammasome is activated under high fructose [100]. Our research group shows that activation of NF-κB signaling and NLRP3 inflammasome are driven by fructose in liver and extrahepatic tissues, causing inflammation, lipid accumulation and insulin signaling impairment in kidney and hypothalamus [101,102,103,104]. Furthermore, ROS generation under fructose-induced hyperuricemia is the crucial factor for podocyte injury by activating p38 MAPK/thioredoxin-interacting protein (TXNIP)/NLRP3 inflammasome pathway [97]. While upregulation of TLR4/myeloid differentiation primary response gene 88 (MyD88) signaling promotes NF-κB signaling in kidney of hyperuricemic mice with high fructose diet [105]. High serum UA also causes inflammation in hypothalamic, vascular endothelium, primary gouty arthritis via acting NF-κB signaling. Pancreatic inflammation may link to elevated serum UA level induced by fructose, since in a rat insulinoma cells, the low-grade pancreatic inflammation is induced by UA [106]. Pancreas islet size and number are increased in fructose-fed rats with impaired morphology and tissue dysfunction [34]. Function disturbance of pancreas islet has potential link with hepatic inflammation. Fructose induces malondialdehyde (MDA), TNF-α and IL-6 levels in pancreas of rats, with CD68-positive cell infiltration consistently, contributing to irregular insulin secretion in pancreas [107]. These observations suggest that dietary fructose may dramatically accelerate tissue and organ inflammation by high UA-induced ROS generation.

UA-induced endothelial dysfunction is another adverse burden in hyperuricemia [108], which may promote insulin resistance and cardiovascular disease under high fructose consumption. Increase in serum UA level, correlated with hypertension and dyslipidemia, increases the risk of cardiovascular disease. Suppression of renal vasodilation may cause kidney dysfunction with increase in urine sodium retention, decrease in renal UA clearance. Elevated UA, insulin and TG in plasma are considered to be closely associated with incident hypertension. Fructose enhances the effects of a high-salt diet on blood pressure by impairing renal reabsorption of sodium in proximal tubule [109]. Hyperactivity of xanthine oxidase (XO) leads to UA production, positively being correlated with elevated serum UA and systolic blood pressure [110]. Inhibition of endothelial NO synthase (eNOS) results in UA-induced vascular insulin resistance and endothelial dysfunction under high fructose consumption [111], possibly leading to the development of hypertension. Fructose exposure activates ROS-mediated NF-κB signaling in human umbilical endothelial cells (HUVECs) [112]. As a result, tissue factor (TF) expression is elevated, which is crucial in plaque formation during atherosclerosis [113].

Hyperuricemia results from deficiency in renal UA excretion, which plays a pathogenic role in fructose-induced kidney injury. It is reported by our group that fructose induces dysregulation of renal organic ion transporters including GLUT9, renal specific transporter (RST), organic anion transporter 1 (OAT1), OAT3, and urate transporter (UAT), which cause abnormal renal UA excretion involved in hyperuricemia and renal dysfunction [114]. Meanwhile, upregulation of renal prostaglandin E_2_ (PGE_2_), a primary mediator of inflammation, is detected in kidney of fructose-fed rats, which may be associated with the dysregulation of renal organic ion transporters [114]. High UA creates arterial stiffness and subsequent renal dysfunction in CKD. Elevation of serum UA induced by fructose activates renin-angiotensin-aldosterone system (RAAS) in perivascular adipose tissue, increases vascular stiffness and causes inflammatory response [115]. Fructose induces chemokine overproduction, such as intercellular adhesion molecule-1(ICAM-1) [116], MCP-1 [117], IL-1β [100], TNF-α and IL-6 [118] in systemic circulation and kidney, causing the progress of CKD [117]. In kidney of fructose-fed rodents, inflammatory response has a close relationship with endothelial dysfunction characterized by high expression of iNOS, COX-2 and ICAM-1 [98,116], as well as fibrosis characterized by increased concentration of glycation end products (RAGE) and α-smooth muscle actin (α-SMA) [99].

Therefore, oxidative stress, inflammatory response and endothelial dysfunction cooperatively impair tissue functions in fructose-induced UA overproduction.

### 3.5. Methylglyoxal (MG)

Fructose upregulates aldolase B to increase MG production in liver, secreting into systemic circulation [119]. This high MG level blocks the allosteric bind of AMP to AMPK. AMPK is a key energy sensor to regulate carbohydrate and lipid in various tissues and organs, including liver, skeletal muscle, adipose and hypothalamus. AMPK activation inhibits acetyl CoA carboxylase (ACC) to reduce malonyl CoA, a substrate for FFA synthesis [120]. Accordingly, FFA oxidation is promoted since malonyl CoA acts as inhibitor of carnitine-palmitoyl-CoA transferase-1 (CPT1). Therefore, the impairment of AMP-sensing capacity of AMPK by MG promotes DNL, leading to the development of fatty liver and insulin resistance under high fructose diet [121,122]. Meanwhile, AMPK suppression gives rise to gluconeogenesis and glucose output, all of which may promote fructose-induced MetS [122].

High fructose consumption induces hypertension possibly by increasing MG level in main aorta and kidney [119,123]. Elevated MG level and upregulation of aldolase B are observed in cultured rat aortic vascular smooth muscle cells [123] and aorta [124] under high fructose level, while aldolase B knockout prevents MG formation in cultured endothelial cells [125]. The promotion of vascular tone involves the upregulation of the high MG-activated renin angiotensin system. Meanwhile, MG induces advanced glycation end-product (AGE) overproduction, NF-κB activation and oxidative stress in vascular smooth muscle cells [119,123]. In fructose-fed rats, high renal MG and renin levels [126] may induce inflammation by activating receptor for AGEs and increasing NF-κB activation [123]. 

## 4. Indirect Dangerous Factors in Tissue and Organ Dysfunctions under High Fructose Consumption

### 4.1. Inflammatory Cytokines

Increasing evidences suggest that fructose-induced MetS is closely associated with chronic inflammation, characterized by elevated peripheral blood mononuclear cells, reduced bone marrow mononuclear cell viability [127], increased systemic inflammation cytokine concentration [127], as well as inflammation signaling activation in local tissues and organs, including liver, adipose, kidney, heart and brain [128].

Adipose tissue secrets adipokines (adiponectin, resistin, apelin and visfatin), hormones (leptin) and chemokines (MCP-1, IL-8, IL-6, IL-1, Ang-II, TNF-α, and IL-10), modulating whole energy homeostasis. Under high fructose consumption, adipose tissue is the key site, giving rise to the secretion of inflammatory cytokines in systemic circulation [129]. Adipose dysfunction can disturb energy expenditure and insulin signaling mainly through inflammatory cytokines [130]. Inflammatory response, accompanied with morphological and functional changes, increased visceral adiposity and fat accumulation, and insulin signaling impairment are detected in adipose tissue of humans or rodents with high fructose diet [29,129]. Endothelial dysfunction induced by fructose is in line with a significant infiltration of macrophages and T cells in perivascular adipose tissue [131]. Local RAAS activation partially gives rise to adipose dysfunction by promoting inflammation, insulin resistance, endothelial dysfunction and vascular stiffness [115].

Long-term treatment of IL-6, IL-1β, or TNF-α is shown to impair insulin signaling [28]. Dysfunction of adipose tissue increases plasma FFA concentration, further inducing insulin resistance [132]. Activation of Akt [11] or TLR4-mediated inflammatory signaling [133] accounts for FFA-induced insulin resistance. Autophagy is shown to be upregulated in adipose tissue of obese humans, the inhibition of which results in secretion of proinflammatory cytokines [134]. Autophagy-associated genes, including autophagy-related gene 7 (ATG7), lysosomal-associated membrane protein 2 (LAMP2) and microtubule-associated protein 1 light chain 3 beta (MAP1LC3β) are downregulated in adipose and liver under high fructose consumption [135]. TNF-α promotes ceramide and FFAs release in systemic circulation [136,137], causing insulin resistance of peripheral tissues and organs [60,138]. TNF-α induces IRS-1(Ser^307^) phosphorylation to decrease insulin sensitivity in adipose tissue [28,139]. A series of serine kinases, including ERK, c-JNK and p38 MAPK [39] can sense lipid metabolites, and inflammatory cytokines in adipocytes and skeletal muscle cells, the activation of which under TNF-α further disturbs the functions of local tissues and organs [139]. IL-6 negatively affects insulin signaling to increase glucose uptake in skeletal muscle and suppress glucose production in liver [140]. The possible mechanism may involve the activation of serine-threonine protein kinase (LKB)1/AMPK/AS160 and JNK-mediated suppression of IRS-1 phosphorylation in skeletal muscle [140]. High fructose diet induces insulin resistance in skeletal muscle, with nuclear translocation of NF-κB 65, and subsequent secretion of IL-6, which is known to be mostly released from skeletal muscle [141]. Correspondingly, expression of inducible NOS (iNOS) and ICAM-1 is changed, suggesting that the possible role of insulin resistance in skeletal muscle may act in an NF-κB-dependent manner [142]. Peroxisome proliferator-activated receptor (PPAR)-δ, one of the most promising pharmacological targets implicated in obesity-associated insulin resistance, is highly expressed in skeletal muscle. Fructose-induced disturbance of PPAR-δ-mediated lipid accumulation and fibroblast growth factor (FGF)-21 production, a myokine in tissue cross-talk, finally induce insulin resistance in skeletal muscle [141]. Fructose also blocks vasodilation in aorta via triggering inflammatory response. Fructose induces overproduction of NO and plasminogen activator inhibitor (PAI)-1 in endothelial cells [143]. NF-κB activation as well as TNF-α and IL-6 secretion impair insulin-triggered endothelial homeostasis partly via activating PI3K/Akt/eNOS and MAPK pathway [143]. Palmitate can induce hepatocytes to release extracellular vesicles in a death receptor 5 (DR5)-dependent manner. EVs induce mRNA expression of IL-1β and IL-6 in mouse bone marrow-derived macrophages, contributing to liver inflammation and injury [144], thereby indicating that hepatic DNL may give rise to systemic inflammatory cytokine secretion. Therefore, TNF-α, IL-1β and IL-6 secretion under high fructose consumption may account for insulin resistance, chronic inflammation and endothelial dysfunction in local tissues and organs.

Fructose consumption also induces psychological stress through inflammatory mechanism [145]. Intracellular inflammatory response is observed in brain and particularly in hypothalamus in MetS [146,147]. Hypothalamus monitors appetite, energy expenditure, carbohydrate and lipid metabolism, and blood pressure. Our group reports that inflammation response in hypothalamus causes local insulin signaling impairment in fructose-fed rats [103]. Fructose feeding induces hippocampal microglia activation through the activation of TLR4/NF-κB signaling, resulting in the reduction of neurogenesis in dentate gyrus of mice. Furthermore, fractalkine (FKN) and its receptor CX3CR1 participate in fructose-induced neuro-inflammation via the activation of TLR4/NF-κB signaling in hypothalamus [148].

Food intake is increased with central administration of fructose via affecting hypothalamic AMPK/malonyl-CoA signaling system to increase food intake in mice [77]. Moreover, fructose activates the hunger signal while depressing the satiety signal by decreasing the serum level of peptide YY3-36 (PYY) and upregulating hunger peptide neuropeptide Y (NPY) mRNA in hypothalamus, showing leptin resistance [149].

### 4.2. Adiponectin

Adiponectin is the most abundant adipokine secreted by adipose tissue. Dysfunction of adipose tissue leads to uncontrolled lipolysis, systemic insulin resistance, ectopic inflammation and lipid accumulation [150], aggravating the development of metabolic disorders. Fructose inhibits the secretion of adiponectin [151] and leptin [151] from adipose tissue into systemic circulation, accompanied with high plasma ghrelin concentration [152]. Adiponectin can reduce apoB and TG production to suppress VLDL release [153]. Decreased adiponectin levels in systemic circulation closely correlates with accumulation of vesical adipose and dysregulation of insulin-stimulated glucose uptake and utilization [154]. Therefore, abdominal and visceral adiposity, reduced insulin-sensitive visceral adipocytes, as well as increased body weight and fat under high fructose feeding [29,155], cause adiponectin secretion reduction [151].

Relevant work reports the crucial role of adiponectin in obesity and liver disease. Serum adiponectin concentration is decreased in rodents with increased influx of neutrophils in liver after high fructose intake, suggesting the possible modulation of neutrophil recruitment [156]. In ketohexokinase (KHK)-KO mice, fructose consumption does not change insulin sensitivity, adiponectin sensitivity and visceral obesity, indicating that the burden of MetS is closely associated with fructolysis [157]. Adiponectin receptor 1 (AdipoR1) and AdipoR2 are expressed in liver and skeletal muscle of humans, while AdipoR2 is mostly expressed in liver of rodents. Genetic variance of AdipoR1 and AdipoR2 genes is associated with liver fat contents in humans [158]. Downregulation or overexpression of AdipoR2 cause or ameliorate the development of liver fibrosis in mice [159]. Adiponectin is known for its anti-inflammatory activity. It reduces TNF-α and induces IL-10 release from Kupffer cells [160], and upregulates chemokine interleukin 8 (CXCL8) in an AdipoR1- and NF-κB-dependent manner in primary human hepatocytes [161]. Adiponectin also blocks CD95-mediated FFA uptake [162] and FFA-induced c-JNK activation, leading to NAFLD development [163]. Adiponectin also reduces liver ROS production via activating superoxide dismutase 1 and catalase [164]. Therefore, hypoadiponection may be one of the contributors to oxidative stress, inflammation response, lipid accumulation and fibrosis in liver under high fructose consumption.

### 4.3. Leptin

Leptin is mainly produced in adipocytes, controlling food intake and energy expenditure. High fructose diet gives rise to leptin resistance in adipose, characterized by down-expression of leptin and leptin receptor (LEPR) in rats [135], possibly affecting autophagy [165]. Stearoyl-CoA desaturase is the rate-limiting enzyme catalyzing monounsaturated FA synthesis. Leptin prevents lipid accumulation and ameliorates insulin sensitivity in liver by downregulating stearoyl-CoA desaturase [166]. Leptin promotes liver fibrogenesis partially by inducing TGF-β1 [167].

On the other hand, high fructose consumption decreases circulating leptin concentration to increase the appetite for over-nutrients intake via affecting hypothalamus function [152]. Decreased ghrelin (major active form of ghrelin, secreted from stomach) and PYY (secreted from lower intestine) levels, increased leptin levels in serum are observed in rats with high fructose consumption [168], cooperatively increasing appetite and food intake. Leptin, ghrelin and PYY are secreted into system circulation and target hypothalamus, where is the appetite center and energy sensor of the whole body. Hypothalamus releases central appetite peptides including NPY and satiety peptide pro-opiomelanocortin (POMC) to increase food intake under fructose consumption [168]. Modulation of these appetite peptides may account for food intake reduction and be part of a defense mechanism against consumption of over nutrient diet [152].

### 4.4. Endotoxin

High fructose consumption-induced MetS correlates with increased intestinal permeability [169], translocation of bacterial endotoxin [170], and intestinal bacterial composition change [171], causing endotoxemia [172]. Elevation of plasma lipopolysaccharide and TNF-α levels, as well as insulin resistance in WAT of rats with high fructose diet, are restored by treatment with antibiotic or faecal samples from control donor rats [171], suggesting intestinal permeability impairment, which is closely associated with systemic or local inflammation response. KHK-C is reported to be expressed in both small bowel and cecum of mice [173]. Fructose feeding increases KHK mRNA expression in duodenum [173]. The acceleration of fructolysis in intestine may cause local inflammation and reduce tight junction protein (occludin and ZO-1) expression in intestine, documenting an increase in intestinal permeability [174]. Meanwhile, high levels of circulating inflammatory cytokines, which are often observed in fructose-fed animals or patients [127,128], may impair intestinal mucosal integrity and induce portal blood endotoxemia [175,176].

Growing evidence supports that increased intestinal permeability participates in fructose-induced MetS, giving rise to the development of NAFLD and chronic inflammation [177]. Fructose-induced endotoxemia activates Kupffer cells via upregulating TLR4/MyD88, which may be partially involved in the development of NAFLD [170], and subsequently trigger NF-κB activation and TNF-α overproduction [178]. Hepatic steatosis and inflammation are significantly ameliorated in TLR4-mutant mice compared with TLR4-WT mice [179]. Knock-out of lipopolysaccharide-binding protein (LBP) partially protects mice from fructose-induced NAFLD by blocking endotoxin from binding to TLR4 in liver [180]. Meanwhile, ROS production also participates in endotoxin-dependent development of NAFLD [181]. Gut-derived endotoxin can trigger hepatic and plasma lipocalin-2 (LCN-2) expression, as it is closely correlated with mitochondrial dysfunction and lipid peroxidation in fructose-induced NAFLD of rats [182]. Endotoxin-triggered inflammatory response in rat aorta is to induce iNOS and cyclooxygenase-2 (COX-2) [183].

## 5. Conclusions

**Figure 1:**
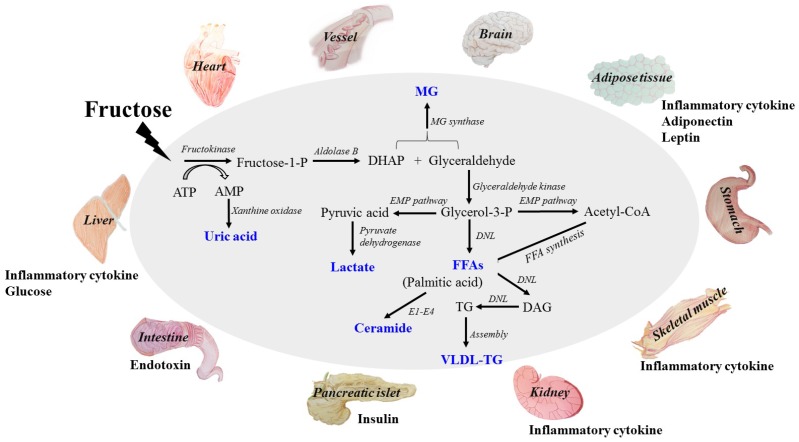
The metabolites of fructose catabolism and the adverse effects of high fructose consumption on tissue and organ functions in a direct and/or indirect manner. Fructose is mainly metabolized in liver to produce glucose, lactate, triglyceride, free fatty acid, uric acid and methylglyoxal. High levels of these metabolites are the direct dangerous factors. These dangerous factors impair the functions of local tissues and organs to overproduce inflammatory cytokine, adiponectin, leptin and endotoxin, which act as indirect dangerous factors. Meanwhile, glucose, insulin and ghrelin contents in system circulation are also disturbed. Fructose and its metabolites directly and/or indirectly cause oxidative stress, chronic inflammation, endothelial dysfunction, autophagy and increased intestinal permeability, and then further aggravate metabolic syndrome with tissue and organ dysfunctions. DHAP, dihydroxyacetone phosphate; TG: triglyceride; FFA: free fatty acid; UA: uric acid; MG: methylglyoxal; VLDL-TG: very low-density lipoprotein-TG. DNL: de novo lipogenesis. E1: Serine palmitoyltransferase; E2: 3-ketodihydrosphingosine reductase; E3: Ceramide synthase; E4: Dihydroceramide desaturase.

Fructose is widely found in natural foods, including fruits, vegetables and honeys, and is added to commercial food additives. Overconsumption of fructose is a risk factor for the epidemic of metabolic syndrome (MetS), with dysfunctions in multiple tissues and organs including liver, adipose, pancreatic islet, skeletal muscle, kidney, heart, brain and intestine. The primary metabolites from fructolysis are produced in liver and secreted into system circulation, directly affecting tissue and organ functions; among these free fatty acids (FFA), uric acid (UA) and lactate play central roles in inducing insulin resistance in systemic and local tissue and organ, as well as causing reactive oxygen species (ROS) overproduction. These dysfunction events consequently lead to secretion of indirect dangerous factors, such as inflammatory cytokine, adiponectin, leptin and endotoxin. These indirect adverse factors give rise to inflammatory response, lipid accumulation, and endothelial dysfunction in local tissues and organs, in addition to the appetite disturbance for food intake, further aggravating the metabolic burden of fructose (summarized in Figure 1). Discussion of these direct and indirect adverse molecules in circulation helps us to uncover the clues for tissue and organ function disturbance and their correlation (Table 1). These adverse effects of high fructose consumption remind us to be cautious about excess fructose intake in our daily diet. More importantly, relevant government departments should make policies about the quality standard and safety of food additives to improve supervisions.

## Figures and Tables

**Table 1:** Pathological changes of major organs and molecular mechanisms of tissue dysfunction under high fructose condition.

Organs Histopathological Changes	Dangerous Factors	Pathological Indexes		Molecular Mechanisms
		↑	↓	
**Adipose tissue** Inflammation response Endothelial dysfunction	FFA UA	ROS production Inflammatory cytokine flux FFA uptake Adiponectin secretion Lipid accumulation Autophagy	Insulin sensitivity Leptin sensitivity Glucose uptake Oxygen availability	PKCθ/IKK-β/c-JNK [39,40,41] IRS/Akt/GLUT4 [60] FATPs/CD36 [70] RAAS [115] LEPR/Stearoyl-CoA desaturase [135,165,166] ATG7/LAMP2/MAP1LC3β [135]
**Brain** Appetite increase Psychological stress	FFA UA MG	ROS production Inflammation cytokine flux Food intake	Insulin sensitivity Leptin sensitivity	TNF-α/AMPK/malonyl-CoA [76,77] NLRP3/NF-κB [95] TLR4/NF-κB, FKN/CX3CR1 [148] PYY, NPY [149]
**Heart/vessel** Hypertrophy Endothelial dysfunction Plaque formation Vascular stiffness	FFA UA	ROS production FFA uptake Vascular tone RAGE production Blood pressure	Insulin sensitivity Glucose consumption Vascular vasodilation	HK/PFK [22] FATPs/CD36 [61] CD36/TLR4/6/IRAK4/1/NLRP3 [67] AMPK/malonyl-CoA [77] XO/eNOS [110,111] PI3K/Akt/eNOS [143]
**Intestine** Increased intestinal permeability	UA	Endotoxin translocation Bacterial composition disturbance Dysregulation of tight junction protein	Insulin sensitivity	SR-BI/ERK/ApoB [80] KHK/Occludin and ZO-1 [173,174]
**Kidney** CKD Endothelial dysfunction	UA MG	ROS production Inflammatory cytokine flux Dysregulation of renal organic ion transporters NO production Urine sodium retention	Insulin sensitivity UA clearance	HK/PFK [22] XO/eNOS [110,111] NLRP3/NF-κB [92,93,94,96] PGE_2_/Organic ion transporters [114] MAPK/TXNIP/NLRP3 [97,100,101,102,103,104,105] TLR4/MyD88/NF-κB [105]
**Liver** Steatosis NAFLD Fibrogenesis Endothelial dysfunction	Lactate FFA DAG Ceramide UA MG	Gluconeogenesis Glucose export ROS production DNL Inflammatory cytokine flux Lipid accumulation Mitochondrial dysfunction VLDL-secretion	Insulin sensitivity Glucose consumption Glucose uptake Oxygen availability	IRS/PI3K/Akt, ChREBP/SCD-1 [11] HK/PFK [22,91] ChREBP/G6Pase [36] Bax/cathepsin B/NF-κB/TNF-α [38] PTP1B/IRS/PI3K/Akt [42] PKC/Akt2/GS/G6Pase/PEPCK [56] SphK1/S1P/NF-κB [61] NOX4/PTP1c [66] SREBP-1c [80] PCSK9/LDLR [82] SR-BI/ERK [88] AMPK/ACC [120] LEPR/ATG7/LAMP2/MAP1LC3β [135] AdipoR1,2/NF-κB/CXCL8 [158,161] CD95, c-JNK [161,162] TLR-4/MyD88 [170] LCN-2 [182]
**Pancreatic islet** Glucose intolerance Increased β-cell mass Irregular insulin secretion	Glucose FFA UA	Inflammatory cytokines flux ER stress Apoptosis	Insulin sensitivity Leptin sensitivity	TR [14] Akt/FoxO1 [15] SREBP-1c/IRS-2/Akt [44] Cideb [48] FFAR1 [49] NF-κB [106]
**Skeletal muscle** Inflammation response Endothelial dysfunction	Lactate FFA Ceramide UA	ROS production FFA uptake Autophagy Inflammatory cytokine flux Lipid accumulation	Insulin sensitivity Glucose uptake Oxygen availability	PI3K/Akt [21] HK/PFK [22] GLUT4 [23,24] FATPs/CD36 [23,61,62] PKCθ/IKK-β/c-JNK [40] LKB1/AMPK/AS160/IRS [140] PPAR-δ/FGF-21 [141] NF-κB/IL-6/iNOS, ICAM-1 [142]

## Abbreviations

ACC	acetyl CoA carboxylase
AdipoR	adiponectin receptor
AGEs	advanced glycation endproducts
AS160	Akt substrate-160 kDa
AMPK	AMP-activated protein kinase
ApoB100	apolipoprotein B100
ATG7	autophagy-related gene 7
Bax	Bcl-2-associated X protein
ChREBP	carbohydrate response element binding protein
Cideb	cell death-inducing DFF45-like effector b
CVD	cardiovascular disease
CPT1	carnitine-palmitoyl-CoA transferase-1
CXCL8	chemokine interleukin 8
CKD	chronic kidney diseases
COX-2	cyclooxygenase-2
DNL	de novo lipogenesis
DR5	death receptor 5
DAG	diacylglycerol
DHAP	dihydroxyacetone phosphate
ER	endoplasmic reticulum
FGF-21	fibroblast growth factor-21
FKN	fractalkine
FFA	free fatty acid
FFAR1	free fatty acid receptor 1
KHK	fructokinase
FATPs	FA transport proteins
FoxO1	Forkhead box protein O1
RAGE	glycation end products
GS	glycogen synthase
G6P	glucose-6-phosphatase
GLUT5	glucose transporter 5
HK	hexokinase
HDL	high-density lipoprotein
HFCS	high-fructose corn syrup
HUVECs	human umbilical endothelial cells
NPY	hunger peptide neuropeptide Y
HIF-1	hypoxia-inducible factor-1
iNOS	inducible NOS
IR	insulin receptor
CAM-1	intercellular adhesion molecule-1
IL	interleukin
IIRAK4/1	IL-1R-associated kinase 4/1
IKK-β	IκB kinase-β
JNK	c-jun *N*-terminal kinase
LEPR	leptin receptor
LCN-2	lipocalin-2
LBP	lipopolysaccharide-binding protein
LDL	low-density lipoprotein
LDLR	LDL receptor
LAMP2	lysosomal-associated membrane protein 2
MDA	malondialdehyde
MetS	metabolic syndrome
MG	methylglyoxal
MAP1LC3β	microtubule-associated protein 1 light chain 3 beta
MCP-1	monocyte chemotactic protein-1
NOX4	NADPH oxidase 4
NLRP3	NOD-like receptor superfamily, pyrin domain containing 3
NAFLD	non-alcoholic fatty liver
NF-κB	nuclear factor kappa B
OAT1	organic anion transporter 1
PYY	peptide YY3-36
PPAR-δ	peroxisome proliferator-activated receptor-δ
PI3K	phosphatidylinositol 3-kinase
PFK	phosphofructokinase
PAI-1	plasminogen activator inhibitor-1
PEPCK	phosphoenolpyruvate carboxykinase
PUFA	polyunsaturated fatty acid
PCSK9	proprotein convertase subtilisin/kexin type 9
PGE2	prostaglandin E2
PKB/Akt	protein kinase B
PKC	protein kinase C
PP1c	protein phosphatase 1c
PTP-1B	protein tyrosine phosphatase-1B
POMC	satiety peptide pro-opiomelanocortin
ROS	reactive oxygen species
RST	renal specific transporter
RAAS	renin-angiotensin-aldosterone system
CD36	scavenger receptor 36
SR-BI	scavenger receptor class B type I
LKB1	serine-threonine protein kinase 1
SGBS	Simpson-Golabi-Behmel syndrome
α-SMA	α-smooth muscle actin
SphK1	sphingosine kinase SphK1
S1P	sphingosine 1-phosphate
SCD-1	stearoyl-coenzyme A desaturase-1
SREBP-1c	sterol regulatory element-binding protein 1c
TR	taste receptor
TXNIP	thioredoxin-interacting protein
TF	tissue factor
TGF-β1	transforming growth factor-β1
TRIB3	tribbles homolog 3
TCA	tricarboxylic acid cycle
TG	triglyceride
TLR4	toll-like receptor 4
TNF-α	tumor necrosis factor-α
T2DM	type 2 diabetes
UAT	urate transporter
UA	uric acid
VLDL	very low-density lipoprotein
WAT	white adipose tissue
XO	xanthine oxidase
